# Lower platelet counts and antiplatelet therapy independently predict better outcomes in patients with head and neck squamous cell carcinoma: a retrospective analysis

**DOI:** 10.1186/s40364-015-0051-2

**Published:** 2015-10-06

**Authors:** Carlo Furlan, Agostino Steffan, Jerry Polesel, Marco Trovo, Carlo Gobitti, Emanuela Vaccher, Diego Serraino, Luigi Barzan, Giovanni Franchin

**Affiliations:** Department of Radiation Oncology, Centro di Riferimento Oncologico (CRO) National Cancer Institute, Via Franco Gallini, 2, 33081 Aviano, Italy; Department of Clinical Pathology, Centro di Riferimento Oncologico (CRO) National Cancer Institute, Aviano, Italy; Department of Statistics and Epidemiology, Centro di Riferimento Oncologico (CRO) National Cancer Institute, Aviano, Italy; Department of Surgery, Centro di Riferimento Oncologico (CRO) National Cancer Institute, Aviano, Italy

**Keywords:** Head and neck cancer, Aspirin, Antiplatelets, Radiotherapy, Coagulation, Platelets, Larynx, Survival

## Abstract

The paper by Rachidi *et al.* suggests that antiplatelet drugs may play a role in ameliorating the clinical outcome in a large series of patients with head and neck cancer managed with either surgery or radiation. Our data, as well as confirming the results observed by the authors, enhance their clinical relevance pointing out the effect of antiplatelet drugs in terms of locoregional control in the setting of patients with advanced head and neck cancer managed with definitive chemo-radiotherapy.

## Previous findings on patients with head and neck cancer exposed to antiplatelet drugs

We read with great interest the article by Rachidi *et al.* [1], which indicated that higher platelet count is associated with a worse survival in head and neck squamous cell carcinoma (HNSCC). In the same cohort the use of antiplatelet medications was associated with a reduced risk of death.

The authors included patients with stage I–IV HNSCC, irrespective of treatment type (surgery, chemotherapy, or radiation).

## Case series report

**Fig. 1:**
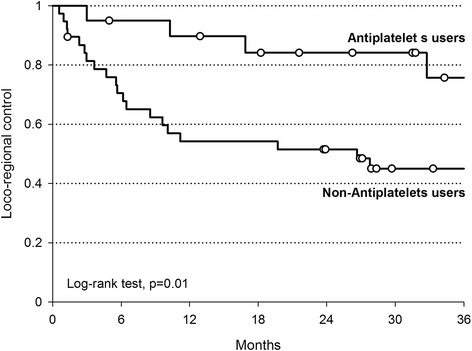
Locoregional control of 58 advanced larynx-hypopharynx cancer patients by use of antiplatelet drugs

We retrospectively reviewed a cohort of 58 consecutive patients with stage III-IV larynx-hypopharynx cancer managed with definitive chemoradiotehrapy at the Centro di Riferimento Oncologico of Aviano, Italy, between 2008 and 2012 (patients characteristics by use of antiplatelet drugs are shown in Table 1). Kaplan-Meier analysis confirmed a survival advantage in patients who were administered with antiplatelet medications, although not significant because of small sample size. At a median follow-up of 31 months, the 3-year survival of patients who were taking antiplatelet drugs (20 patients) was 69 % vs 54 % for those who were not taking (*p* = 0.12). Interestingly, our data also showed a significant difference in locoregional control (LRC) between patients who were and were not taking antiplatelet medications during radiotherapy (3-year LRC resulted 76 % and 45 %, respectively, *p* = 0.01 - Fig. 1).

**Table 1:** Baseline socio-demographic and clinical characteristics of 58 larynx-hypopharynx cancer patients, according to regular antiplatelets use

	Non-Antiplatelets users		Antiplatelets users		Fisher exact test
	N	(%)	n	(%)	
Sex					
Men	34	(89.5)	16	(80.0)	*p* = 0.43
Women	4	(10.5)	4	(20.0)	
Age (years)					
<65	28	(73.7)	5	(25.0)	*p* <0.01
≥65	10	(26.3)	15	(75.0)	
Tobacco smoking					
Never	5	(13.2)	4	(20.0)	*p* = 0.70
Ever	33	(86.8)	16	(80.0)	
T status					
T2–T3	27	(71.0)	20	(100.0)	*p* = 0.01
T4	11	(29.0)	0	(0.0)	
N status					
N0	11	(29.0)	8	(40.0)	*p* = 0.56
N1–N3	27	(71.0)	12	(60.0)	
Stage					
III	13	(34.2)	15	(75.0)	*p* <0.01
IV	25	(65.8)	5	(25.0)	
Platelet count at radiotherapy beginning (cells/μL)					
<250.000	19	(51.3)	11	(57.9)	*p* = 0.78
≥250.000	18	(48.7)	8	(42.1)	
Locoregional recurrence					
No	17	(44.7)	16	(80.0)	*p* = 0.01
Yes	21	(55.3)	4	(20.0)

Since antiplatelet treatment was significantly associated to age and tumor stage, subgroup analysis were also conducted. The same effect was observed both patients aged <65 year (3-year LRC was 80 % vs 40 % for those who were and were not taking antiplatelet drugs), and in the those aged ≥65 year (74 % vs 58 %, respectively), as well as in patients with stage III (75 % and 54 %, respectively) and stage IV cancer (74 % and 40 %, respectively).

Rachidi and colleagues [1] reported stronger effect of antiplatelet use on OS in patients with thrombocytosis. In our study, we also found a greater advantage from antiplatelet use among patients with platelet count >250.000/μL (Table 1) in both OS (3-year OS: 63 % vs 43 % in antiplatelet users and non users, *p* = 0.50) and LRC (3-year LRC : 71 % vs 18 %, *p* = 0.02).

## Comment

Our results confirmed that the use of antiplatelet medications modify outcomes in patients with HNSCC. LRC is recognized as a surrogate endpoint of survival for patients with locally-advanced HNSCC [2], and our findings concerning LRC may be translated into survival at longer follow-up, confirming the results published in the paper. Moreover, these elements concerning the effects on LRC suggest that antiplatelet medications may have a role in enhancing the efficacy of radiation for HNSCC and indicate the strong need for a randomized clinical trial.

## Abbreviations

HNCSS: Head and neck squamous cell carcinoma
LRC: Locoregional control
